# Neurodevelopmental Trajectories Following Prenatal Alcohol Exposure

**DOI:** 10.3389/fnhum.2021.695855

**Published:** 2022-01-04

**Authors:** Eileen M. Moore, Yingjing Xia

**Affiliations:** Center for Behavioral Teratology, San Diego State University, San Diego, CA, United States

**Keywords:** prenatal, alcohol, FASD, fetal alcohol spectrum disorder, brain, development, trajectory, MRI

## Abstract

Prenatal alcohol exposure (PAE) interferes with neurodevelopment. The brain is particularly susceptible to the adverse consequences of prenatal alcohol exposure, and numerous studies have documented changes to brain anatomy and function, as well as consequences for cognition, behavior, and mental health. Studies in typically developing individuals have shown that the brain undergoes dynamic developmental processes over an individual’s lifespan. Furthermore, magnetic resonance imaging (MRI) studies in other neurodevelopmental and psychiatric disorders have shown that their developmental trajectories differ from the typical pattern. Therefore, to understand long-term clinical outcomes of fetal alcohol spectrum disorders (FASD), it is necessary to investigate changes in neurodevelopmental trajectories in this population. Here we review studies that have used MRI to evaluate changes in brain structure and function over time *via* cross-sectional or longitudinal methods in individuals with PAE. Research demonstrates that individuals with PAE have atypical cortical and white matter microstructural developmental trajectories through childhood and adolescence. More research is needed to understand how factors such as sex and postnatal experiences may further mediate these trajectories. Furthermore, nothing is known about the trajectories beyond young adulthood.

## Introduction

Alcohol can interfere with the development of an embryo/fetus and produce a wide range of adverse effects. The term fetal alcohol spectrum disorders (FASD) refers to the full range of adverse outcomes following prenatal alcohol exposure (PAE), including dysmorphism, pre- and/or postnatal growth restriction, and neurobehavioral deficits. FASD is relatively common. A recent meta-analysis estimated that across the globe, 630,000 children are born with FASD every year and, out of 187 countries for which there is data available, 76 have an estimated prevalence of FASD that exceeds 1% (Lange et al., 2017).

The brain is especially vulnerable to PAE. Numerous studies have shown the effect of such exposure on cognition, behavior, and mental health (reviewed in Weyrauch et al., 2017; Mattson et al., 2019). Cross-sectional studies have found neuroanatomical changes in individuals with PAE, including global white-matter volume reduction and decrease in gray matter in specific brain regions (Archibald et al., 2001; Sowell et al., 2001). However, relatively fewer studies have investigated how PAE impacts the development of the brain over time. In typically developing individuals, different brain tissue types and structures undergo distinct developmental trajectories from prenatal stages through adulthood (Giedd and Rapoport, 2010). Past research has shown that developmental disorders differentially impact the neurodevelopmental trajectories of brain structures, and these changes in trajectories could predict long-term clinical outcomes (Giedd and Rapoport, 2010). As PAE disrupts the earliest stages of brain development and can have cascade effects on subsequent developmental processes, it is pertinent for researchers to understand whether PAE also differentially impacts neurodevelopmental trajectories and whether this may contribute to our understanding of the clinical outcomes of FASD. Here we review studies that evaluate the impact of age on brain structure and function, highlight how neurodevelopmental trajectories may differ for individuals with PAE, and discuss areas in need of further research. A summary of the research findings relating to neurodevelopmental trajectories following PAE is available in Table 1.

**Table 1:** Summary of studies examining relations between prenatal alcohol exposure and age on neurodevelopment.

Study	n, PAE	n, CON	Age (years)	Study design	Site	Imaging method	Findings
Gautam et al. (2014)	25	16	6.2–17.6	L	CIFASD: Los Angeles, CA	sMRI	No significant age by status interaction in white matter volume was found in any of the regions investigated
Gautam et al. (2015a)	75	64	7.1–15.9	L	CIFASD: Los Angeles, CA; San Diego, CA; Cape Town, South Africa	sMRI	Those with PAE showed a linear trajectory in right and left transverse temporal regions, while the controls showed no significant age related changes.
Hendrickson et al. (2018)	58	52	6.0–17.0	L	CIFASD: Minneapolis, MN; Los Angeles, CA ; San Diego, CA, Atlanta, GA	sMRI	The control subjects showed a more curvilinear cortical developmental trajectory, while the subjects with PAE showed closer to a linear trajectory.
Lebel et al. (2012)	70	63	5.7–15.9	L	CIFASD: Los Angeles, CA ; San Diego, CA ; Cape Town, South Africa	sMRI	For total gray matter volume, the controls had a more quadratic developmental trajectory; while the subjects with PAE showed a more linear decline, although the age by group interaction was not significant. In posterior brain regions, the controls showed more curved developmental trajectories compared to the more linear trajectories in the subjects with PAE.
Treit et al. (2014)	11	12	5.7–14.4	L	Alberta, Canada	sMRI	No significant age by group interactions were found in terms of volume. The mean cortical thickness decreased with age in the control group but not in those with PAE. Region specific analyses revealed that the differential developmental trajectories concentrated in medial frontal and parietal regions.
Treit et al. (2013)	17	27	5.4–11.8	L	Alberta, Canada	sMRI, DTI	The control group showed age related increases in total brain, white matter, globus pallidus, and amygdala volumes while those with PAE did not. MD decreased more steeply over time in the group with PAE than in the control group.
Gautam et al. (2015b)	49	48	6.0–16.0	L	CIFASD: Los Angeles, CA; San Diego, CA; Cape Town, South Africa	fMRI	Significant group by time interactions showed that the changes in activation pattern over time were different for the group with PAE as compared to controls. For the 0-back condition, the activation increased in controls from time 1 to time 2 but decreased in those with PAE.
Infante et al. (2015)	30	19	13–16	C	San Diego, CA	sMRI	The group with PAE showed decreased gyrification with age in the right frontal, occipital, left frontal and parietal cortices; the controls showed increased gyrification with age in right occipital cortices.
Inkelis et al. (2020)	107	56	13–30	C	Seattle, WA	sMRI	The group with PAE showed an initial increase in volume that peaked in early adulthood, followed by a decrease in volume later in life in the corpus callosum and caudate, as well as a linear decline in cerebellar volume.
Nardelli et al. (2011)	28	56	6.0–17.0	C	Edmonton, Alberta, Canada	sMRI	No significant age-group interactions were found for intracranial volume, white matter volume, cortical gray matter volume, and deep gray matter volume.
Rajaprakash et al. (2014)	36	52	8.1–15.6	C	Toronto, Ontario, Canada	sMRI	No sigificant age by group interactions were found in the cortical thickness and surface area.
Zhou et al. (2011)	33	33	6.0–30.0	C	Alberta, Canada	sMRI	No sigificant age by group interactions were found in the cortical thickness.
Zhou et al. (2018)	78	79	5.5–18.9	C	Vancouver, British Columbia, Canada; Alberta, Canada; Manitoba, Canada; Kingston, Ontario, Canada	sMRI	No age by group interactions were observed for volumes. Cortical thinning trajectories were also similar across groups.
Treit et al. (2017)	70	74	5.0–32.0	C	Alberta, Canada	sMRI, DTI	The control group showed a steeper increase in FA for the SLF and ILF compared to those with PAE. In contrast, individuals with PAE showed a steeper increase of FA in the genu compared to controls. Steeper increases in white matter and amygdala volumes were also observed in those with PAE compared to controls. Cortical thinning was slower in those with PAE vs. controls for the gyrus rectus.
Uban et al. (2017)	31	30	9.3–15.5	C	CIFASD: Minneapolis, MN, Los Angeles, CA	DTI	In control boys, FA increased with age in the cingulum and optic radiation; however, this was not observed for boys with PAE.
McLachlan et al. (2019)	10	14	7.0–18.0	C	Vancouver, British Columbia, Canada	MWF	In those with PAE, MWF increased with age in the corpus callosum and right minor forceps, while in controls the MWF did not significantly correlate with age.

*Note: FA, fractional anisotropy; MD, mean diffusivity; SLF, superior longitudinal fasciculus; ILF, inferior longitudinal fasciculus; PAE, prenatal alcohol-exposed; CON, control; L, longitudinal; C, Cross-sectional; sMRI, structural magnetic resonance imaging; fMRI, functional magnetic resonance imaging; DTI, diffusion tensor imaging; MWF, myelin water fraction*.

As some studies reviewed here employed longitudinal designs while others used cross-sectional designs, it may be beneficial to briefly discuss the definition of these two designs and their methodological strength and weakness. In a cross-sectional design, for the specific outcome variable, each subject is only measured at one single time point (Baltes, 1968; Kraemer et al., 2000). In contrast, a study utilizing a longitudinal design measures the same outcome variable at multiple time points from each subject (Baltes, 1968; Kraemer et al., 2000). It is important to note that abundant caution should be applied when one attempts to make inferences about developmental changes from a cross-sectional study. Compared to longitudinal studies, cross-sectional studies may produce misleading inferences due to various limitations, such as unreliable measurement errors over time, non-parallel trajectories, random-time sampling, among others (Kraemer et al., 2000). The majority of longitudinal studies reviewed herein were part of the Collective Initiative on Fetal Alcohol Spectrum Disorders (CIFASD), which is a consortium of clinical and basic experimental scientists seeking to further our understanding of FASD. The longitudinal CIFASD MRI studies included subjects tested at different sites using standardized procedures. In addition to MRI, subjects completed a dysmorphology exam, neurobehavioral testing, and/or 3-D facial imaging (Mattson et al., 2010).

## Total Brain Size

One of the most common and robust magnetic resonance imaging (MRI) findings in the FASD literature is reduced overall brain size (Moore et al., 2014). A longitudinal study by the CIFASD found that not only did the children with PAE have smaller total brain volumes than controls, but they also exhibited atypical trajectories for total brain volume (Lebel et al., 2012). Specifically, when two groups were analyzed separately, the group with severe PAE showed a more linear developmental trajectory as compared to the more pronounced quadratic curve seen in the controls (Lebel et al., 2012). However, the age-by-group interaction term was not significant when the two groups were compared together (Lebel et al., 2012). Other longitudinal studies have reported that trajectories in alcohol-exposed and control children appear to be similar when evaluating total brain volumes (Treit et al., 2013, 2014, 2017). This lack of difference in age-trajectories in total brain volume when directly comparing across groups is not necessarily surprising. Studies have shown that different areas of the brain have distinct developmental trajectories in typically developing children, thus, a rough global evaluation might not reflect differences in trajectories seen in specific regions of interest (Giedd and Rapoport, 2010). Thus, it is important to examine the developmental trajectories of specific brain tissue types and regions in order to fully evaluate the potential atypical brain development patterns in FASD.

## Cortex

Several studies have examined cortical development using either cross-sectional or longitudinal designs, and have measured several different cortical indices, including volume, thickness, surface area, and gyrification. Results of cross-sectional studies are mixed, but the longitudinal studies are generally in agreement that the developmental changes in the cortex across childhood and adolescence differ between children with PAE as compared to typically developing controls.

### Cortical Volume

As part of the CIFASD, Lebel et al. (2012) showed differential volume trajectories of several posterior regions of the cortex in a longitudinal analysis. This study collected MRI scans at three sites (Los Angeles, California; San Diego, California; and Cape Town, South Africa). One hundred thirty-three children participated in at least two MRI scans, and ranged in age between 5.7 and 15.9 years at the first scan, with the second scan collected approximately 2 years later. Volumes of 68 cortical regions of interest were calculated and compared between children with heavy PAE and typically developing controls. Group differences in developmental trajectories were found in several posterior regions of the cortex, including the bilateral inferior parietal, left superior parietal, and lateral occipital, as well as the right postcentral and banks of the superior temporal regions. In controls, an inverted U-shaped trajectory of volume change was observed, whereas the alcohol-exposed group demonstrated a relatively linear volume decline from childhood through adolescence. For a subset of individuals (*n* = 38), the majority of whom were South African, information on the quantity of alcohol exposure was available. A positive relationship between alcohol exposure level and volume change across time was noted in the left posterior cingulate cortex; whereas negative relations were noted in the right transverse temporal and left parahippocampal cortices. Thus, the quantity of alcohol exposure may also impact the developmental trajectory. Furthermore, sex differences in the developmental trajectories of the left precentral, superior parietal, and supramarginal regions were observed in controls, where males had a longer period of volume increase as compared to females. This sex-effect was not observed in children with PAE, which perhaps indicates an attenuation in the sexually dimorphic cortical volume trajectories for those with FASD.

A more recent longitudinal CIFASD study of cortical development in children with PAE used a whole-cortex, vertex-wise approach to examine cortical development in a separate sample of children (Hendrickson et al., 2018). Subjects were aged 6–17 years at the time of their first scan, with a second scan collected approximately 2 years later. Data were collected from 110 children at four CIFASD sites across the United States (Minneapolis, Minnesota; Los Angeles, California; San Diego, California; and Atlanta, Georgia). When cortex volume development was modeled as a quadratic function, no differences in trajectory were observed.

However, when it was modeled as a linear function, significant group differences in the trajectories were noted in 10 clusters within frontal, temporal, and posterior cortical regions. The children with alcohol-exposure showed a more pronounced decrease in percent change compared to the controls, which leads to a larger volume discrepancy between the groups during adolescence as compared to younger ages.

While both the CIFASD longitudinal studies support group differences in developmental volume change, albeit differing in the affected brain regions, another study found no significant between group differences in age-related volume change when examining cortical development in children with FASD (Rajaprakash et al., 2014). Two differences between the samples used in these studies might explain this discrepancy in results. First, the study by Rajaprakash et al. (2014) was cross-sectional and had a much smaller sample size than the CIFASD reports, which may lead to a lack of power in discerning trajectory differences. Second, the study by Rajaprakash et al. (2014) only included children with a diagnosis of alcohol-related neurobehavioral disorder (ARND). Children with ARND lack alcohol-induced facial dysmorphology but have behavioral and/or cognitive deficits associated with PAE. In contrast, the CIFASD studies evaluated children with heavy PAE, which included children with and without alcohol-induced facial dysmorphism. Indeed, the CIFASD samples included a portion of children with fetal alcohol syndrome, who often have microcephaly. It is possible that the presence of facial dysmorphism and/or more severe neuroanatomical changes from PAE may influence trajectories. Indeed, in the CIFASD study, when examining the relationship between cortex volume rate-of-change and facial dysmorphology, Lebel et al. (2012) found that facial dysmorphology was related to volume change in 22 mostly frontal lobe regions in children with alcohol-exposure. Generally, worse dysmorphology was associated with less volume change, perhaps indicating poorer neuroplasticity in the children with alcohol exposure and facial dysmorphology.

### Cortical Thickness

The longitudinal CIFASD study by Lebel et al. (2012) also examined group differences in the developmental trajectory of cortical thickness using linear and quadratic models. Eight cortical clusters with group differences in linear trajectories and three with different quadratic trajectories were identified, located in the parietal, temporal, and frontal cortices. Generally, the children with alcohol exposure showed flatter change or more pronounced decreases with development, which resulted in bigger group discrepancies during adolescence as compared to younger ages; although there were some regions in the parietal cortex where the reverse pattern was observed. Treit et al. (2014) also examined developmental change in cortical thickness, using a mixed-models analytic approach in a longitudinal but smaller sample of children aged 5.7–15.4 years. The children were scanned two times 2–4 years apart. This report found that across childhood and adolescence, the control subjects demonstrated significant developmental cortical thinning over time, but the subjects with alcohol-exposure did not. Further analysis showed that the developmental effects were driven by a steeper rate of cortical thinning in frontal and parietal brain regions in the controls than in the children with alcohol exposure. Later the same research group conducted another analysis of the developmental trajectories of cortical thickness with a larger cross-sectional sample and found a significant group by age interaction that showed a steeper decrease in cortical thickness in the gyrus rectus in the control group as compared to the children with PAE (Treit et al., 2017). Although the studies discussed above indicate atypical cortical thickness trajectories in the children with alcohol-exposure (Treit et al., 2014, 2017; Hendrickson et al., 2018), it is worth noting that three other cross-sectional studies have failed to find significant group differences in the relationship between age and cortical thickness (Zhou et al., 2011, 2018; Rajaprakash et al., 2014). Cortical thickness measurements appear to be particularly sensitive to methodological issues (e.g., scanners, recording parameters, analysis tools, and head motion) that can influence interpretation, which might contribute to the inconsistencies in the current literature (Walhovd et al., 2017). More research is needed to determine how PAE may be related to cortical thickness.

### Cortical Surface Area

To date, there is no evidence for atypical cortical surface area trajectories in children with PAE. Rajaprakash et al. (2014) evaluated surface area in their study of cortical morphology, and while they found a smaller average surface area in their sample of children with ARND, they did not observe differences in the relationship between age and surface area in those with alcohol exposure as compared to controls. Hendrickson et al. (2018) did not find differences between children with alcohol exposure and controls in cortical surface area, and therefore did not continue further analyses on this metric.

### Cortical Gyrification

Gyrification measures provide a summary of how folded the cortex is. Changes in gyrification can be indicative of abnormal cell migration during prenatal development. Additionally, there is a link between a smoother cortex and lowered IQ in individuals with FASD (Hendrickson et al., 2017). While the majority of cortical folding occurs prenatally, gyrification may also change postnatally. Only one cross-sectional study (Infante et al., 2015) and one longitudinal study (Hendrickson et al., 2018) have examined the impact of age on cortical gyrification yet. Both studies used the Local Gyrification Index (LGI), which is a metric that quantifies the degree to which cortex is buried within the sulcal folds vs. exposed on the surface. A larger LGI score indicates more extensive folding, whereas a smaller score indicates more limited folding. Infante et al. (2015) examined adolescents with heavy PAE, age 13–16 years, and found four clusters in frontal, temporal, and posterior areas that showed group differences in linear relations between age and LGI. In the group with alcohol exposure, the younger adolescent subjects displayed more extensive folding whereas the older adolescent subjects displayed less extensive folding. However, controls showed either the opposite pattern or no age-associated changes. Hendrickson et al. (2018) modeled both the linear and quadratic effects of age in longitudinal data and with a larger age-range (6–17 years). They found age-by-group interactions in clusters across the bilateral cortex. Linear age-by-group interactions were found in seven clusters within the frontal, temporal, and parietal lobes; and quadratic age-by-group interactions were identified in 10 clusters across all four lobes. Similar to what these authors had observed with cortical thickness trajectories, in the majority of clusters, the adolescents with PAE showed either blunted change or more pronounced decrease over time as compared to controls, although the more pronounced decrease was observed for controls in specific areas of the parietal and temporal cortex. Thus, the evidence to date indicates that children with PAE show atypical changes in gyrification across childhood and adolescence.

## Subcortical

The CIFASD has also evaluated age-trajectories in subcortical structures (Gautam et al., 2015a). Children at three sites (Los Angeles, California; San Diego, California; and Cape Town, South Africa; *N* = 139) completed a longitudinal MRI study. Children were aged 7.1–15.9 years at first scan, and the second scan was approximately 2 years later. This study found that while subcortical structures increased in volume across time in both the children with PAE and controls, there was no difference in the trajectories.

In contrast, cross-sectional studies suggest atypical trajectories in specific subcortical regions during distinct age periods in individuals with PAE. One study analyzed relations between age and subcortical volumes (hippocampus, amygdala, thalamus, caudate, putamen, and globus pallidus) by comparing three age groups: young children (6–9 years), early-adolescent (10–13 years), and older-adolescent (14–17 years) subjects (Nardelli et al., 2011). They found that only in the youngest two age groups did children with PAE demonstrate smaller volumes of the caudate and putamen, whereas the amygdala was smaller only in the oldest subgroup (Nardelli et al., 2011). However, these authors also reported that they found no significant interactions between group and age when age was modeled as a continuous variable. Treit et al. (2017) also evaluated volumes of subcortical structures across a larger age range (5–32 years) in a cross-sectional study and found trajectory differences in the amygdala. In their study, the control group demonstrated a steeper increase in amygdala volume compared to the individuals with PAE, but trajectory differences were not observed in other subcortical structures. Inkelis et al. (2020) examined subcortical regions of interest in a cross-sectional analysis of archival data with adolescents and young adults (age 13–30 years) and found evidence of atypical maturation patterns. This MRI data was collected in conjunction with Streissguth and colleagues’ prospective longitudinal study of individuals with PAE (see Streissguth, 2007 for review). Inkelis et al. (2020) evaluated volumes of regions known to be particularly sensitive to the impact of PAE, including the corpus callosum, basal ganglia, and cerebellum (Roebuck et al., 1998) in adolescents and adults with FASD. Though their analysis showed no significant differences in age-related volume changes between groups in the putamen or pallidum, differential relations between age and volume were found for the corpus callosum, caudate, and cerebellum. Compared to the linear increase in volume in the control sample, for those with FASD, the brain regions demonstrated an initial increase in volume that peaked in early adulthood, followed by a subsequent decrease in volume.

Thus, it is possible that for some subcortical structures, the children with alcohol exposure have a more modest increase in volume with age across childhood and adolescence than typically developing children, and potential volume reduction after adolescence. However, the longitudinal CIFASD sample did not find evidence of such effects. Caution needs to be observed when drawing conclusions about trajectories from cross-sectional studies. Additional longitudinal MRI studies will be necessary to understand how PAE relates to developmental trajectories of subcortical structures, especially for age ranges beyond adolescence.

## White Matter

A number of studies have evaluated associations between PAE and white matter trajectories, and how white matter development is related to cognitive development. Two main methodologies have been used: evaluation of gross white matter development with volume measurement, and examination of white matter microstructure with diffusion tensor imaging (DTI). Additionally, one recent study used myelin water fraction (MWF) to evaluate associations between PAE and the developmental trajectory of myelin.

### Volume

Several studies have evaluated whether there may be developmental differences across childhood and adolescence in white matter volume (Gautam et al., 2014; Treit et al., 2017; Zhou et al., 2018). Treit et al. (2017) looked at the total white matter in a group of individuals aged 5–32 years (*N* = 144). In this cross-sectional study, they found that the controls demonstrated steeper developmental increases in volume across this age range than did those with PAE. However, two other cross-sectional studies using samples with smaller age ranges (5.5–18.9 years; 6–17 years) found that white-matter volume increased at a similar rate in both the group with prenatal alcohol exposure and the controls (Gautam et al., 2014; Zhou et al., 2018). Among these two studies, Gautam et al. (2014), as part of CIFASD, also evaluated whether white matter volumes predicted cognitive performance differently across groups through childhood and adolescence in several regions of interest, including frontal and parietal lobes, and the corpus callosum. While developmental changes in white matter volume did not relate to cognitive scores in control subjects, developmental increases in total white matter volume, frontoparietal areas and the corpus callosum across childhood and adolescence predicted attention scores in those with PAE (Digit Span Forward). Additionally, they noted that developmental volume increases in the corpus callosum also predicted performance in a free recall of a word list after a 20-min delay (California Verbal Learning Test—Children’s Edition).

A CIFASD longitudinal study examined group differences in the developmental trajectory of white matter volume between children with PAE and the controls and explored how white matter development relates to cognition (Gautam et al., 2014). As noted previously, 139 subjects across three sites completed their first MRI scan at age 7–17, and their second approximately 2 years later. The authors noted that, after a false discovery rate correction for multiple comparisons, bilateral transverse temporal white matter volumes showed significantly different developmental trajectories across groups. The children with alcohol exposure demonstrated a linear volume increase over time, whereas no volume change was observed in controls. They also explored group differences in relations between white matter volume change and cognition and found several differential relationships in those with PAE vs. controls. However, the authors cautioned that the analyses were exploratory and would not be considered significant after a correction for multiple comparisons. Nevertheless, they reported that white matter volume change in the right inferior and superior temporal areas differentially predicted arithmetic scores, left caudal middle frontal volume change differentially predicted parent-reported attention problems, and right pallidum and pars triangularis volume change predicted parent-reported executive function scores. Specifically, larger white matter volume increases over time related to better arithmetic scores, worse attention problems, and improved executive function in the children with PAE, while these relations were either not present or opposite in the control group.

### Diffusion Tensor Imaging

White matter microstructure can be examined with diffusion tensor imaging (DTI), which may provide additional information about neurodevelopment changes associated with PAE. DTI measures differences in water diffusion. Restriction in water molecule movement is used to infer the underlying white matter characteristics based on the assumption that more myelination would lead to more restriction (Assaf and Pasternak, 2008). There are several common DTI metrics for this purpose: fractional anisotropy (FA) is the normalized standard deviation of the diffusivities along with three directions and provides a summary measure of white matter microstructure, whereas mean diffusivity (MD) is the average of the three directional diffusivities.

As part of the CIFASD study, Uban et al. (2017) investigated PAE-related, developmental sex differences in a sample of 61 children (age range: 9.3–16 years) using DTI. They compared children with PAE with a carefully age-matched control group. The results showed altered developmental trajectories in white matter tracts in boys but not in girls. Specifically, FA for cingulum and optic radiation increased with age for the controls but not in boys with PAE. Uban and colleagues noted that the control boys demonstrated a positive association between FA and testosterone levels, which is consistent with the literature; however, this relation was not present in boys with PAE. This finding may suggest a sex-differential delay in white matter maturation during puberty, possibly linked to changes in testosterone’s influence on brain development, in children and adolescents with PAE.

In a longitudinal DTI study comparing children aged 5.4–11.8 years at first scan (with 1.8–4.2 years between scans; 17 PAE vs. 27 controls), group differences in the developmental trajectories of 11 white matter tracts were examined (Treit et al., 2013). Across development, FA increased in white matter tracts for both groups, although the body of the corpus callosum, the inferior longitudinal fasciculus (ILF), and the uncinate fasciculus did not show significant age–related increases in the group with PAE that were seen in controls. Significantly different relations between MD and development were noted between groups in the superior-fronto occipital (SFO), inferior-fronto occipital (IFO), and superior longitudinal (SLF) fasciculi. In all tracts, the group with PAE showed a greater decrease in MD with age compared to controls. A few years later, this same group conducted a cross-sectional study using a larger sample of children and young adults (age 5–32 years; *N* = 144; Treit et al., 2017). In contrast to their prior report, they did not see significantly different developmental trajectories of MD. However, they found that the increases of FA with age in ILF and SLF were steeper in the controls compared to these of the group with PAE, whereas the opposite pattern was seen for the FA of the genu. While both reports indicate atypical trajectories of white matter microstructure, more research is needed to characterize this trajectory.

In their longitudinal report, Treit et al. (2013) also evaluated how the developmental change in white matter microstructure was related to cognition. They found that greater reductions in MD over time in the SFO and SLF predicted larger gains in reading performance in the children with PAE, but no such relation was present in controls [as measured by the Woodcock reading mastery test-revised (WRMT-R) Word ID score]. Additionally, greater MD reductions in the SFO predicted larger gains in receptive vocabulary in the children with PAE [as measured by the Comprehensive receptive and expressive vocabulary test (CREVT) receptive score].

### Myelin Water Fraction (MWF)

Myelin Water Fraction is an *in vivo* quantitative assessment of brain myelin content. One study has investigated the developmental impact of PAE on the microstructure of white matter using MWF in a sample of 24 participants (10 with PAE and 14 controls; McLachlan et al., 2019). The results of this study showed that the MWF for the frontal linked regions of the corpus callosum and right minor forceps increased with age in those with PAE but not in the control group, although mean MWF was comparable in white matter regions across groups. This altered developmental trajectory in myelin may suggest a “catch-up” for early developmental delays in those with PAE.

## Functional MRI

To date, one study has compared developmental trajectories in children with PAE to controls using task-based functional MRI (fMRI; Gautam et al., 2015b). As part of the CIFASD, children with and without heavy PAE underwent functional MRI as they completed a visuospatial N-back task. Children were ages 7–14 at the first scan and completed a second scan approximately 2 years later. Sixty-one children completed scans in Cape Town, South Africa; and the primary analyses occurred on this group. Scans were also collected in Los Angeles and San Diego, California (sample sizes were 20 and 16 children, respectively) and these data were used to confirm results from the South Africa sample. The task included three blocks of 0-back, and two blocks each of 1- and 2-back conditions, as well as a baseline condition that included the initial fixation period before the first task block. In the 0-back, children were asked to press a button each time a dot appeared in the center of the visual field. For 1-back, children had to press a button whenever a dot appeared twice in a row in the same location. There was also a 2-back condition; however, accuracy was poor for most of the participants and therefore it was excluded from analyses. The primary findings were in the visuospatial attention blood oxygen level dependent (BOLD) activation developmental patterns, which were derived from 0-back > baseline and 1-back > baseline contrasts. Within group results revealed that the controls had significant increases in BOLD activation intensities over time for both the 0-back and 1-back (>baseline) contrasts; however, the group with PAE did not demonstrate any significant increases in activation in either contrast. Furthermore, the group with PAE showed decreases in activation over time in the parietal cortex and part of the right cerebellum for the 0-back (>baseline) condition. Significant group-by time interactions were found in frontal, temporal, parieto occipital, hippocampal, and thalamus areas for the 0-back (>baseline condition). This suggests that processes underlying cognitive functions differentially mature in the children with PAE and controls, and it is interesting to note that many of the regions identified in this functional study overlap with those identified in structural studies. More studies using fMRI to assess developmental changes in the brain are necessary to better understand how the cognitive deficits may be linked to atypical neurodevelopmental trajectories.

## Future Directions

Studies have noted atypical neurodevelopmental trajectories in individuals with PAE. Prior literature has shown that neurodevelopmental trajectories differ between males and females. Unfortunately, sex differences in the developmental trajectories for those with PAE have not been adequately assessed. Only one longitudinal study to date has modeled the interactions of group, sex, and age in their analysis (Lebel et al., 2012) and noted areas in the left hemisphere where sexually dimorphic cortical trajectories may be attenuated for those with PAE. This finding replicates previous studies that have noted attenuated sex-differences in the brain among youth with PAE (Moore et al., 2016; Treit et al., 2017; Inkelis et al., 2020). Moreover, one cross-sectional study showed altered white-matter developmental trajectories in males but not in females (Uban et al., 2017). As the brain matures differently across sexes in typically developing children (De Bellis et al., 2001; Kaczkurkin et al., 2019), it is possible that PAE impacts neurodevelopmental trajectories differently across the sexes. Therefore, it is important that future studies examine sex as an independent factor that could interact with prenatal exposure history to predict developmental differences in brain structure and function.

Another important avenue for further research is examination of other factors previously shown to influence neurodevelopment. Research has shown that a number of experiences can mediate neurodevelopment, including socioeconomic status (Piccolo et al., 2016), childhood maltreatment (Paquola et al., 2016, 2017), and psychiatric disorders (Shaw et al., 2010; Ball et al., 2019; Harrewijn et al., 2021), among many others. Few studies to date have matched the typically developed controls with the PAE group on these contextual factors. Streissguth et al. (2004) showed that adverse life experiences and lack of access to appropriate services predicted unfavorable outcomes for those with FASD, while early identification/diagnosis, access to services, and a stable/nurturing home were protective. Moreover, besides the factors mentioned above, individuals with FASD are more likely to develop secondary conditions as a result of the condition that they are born with, such as mental health problems and alcohol problems (Moore and Riley, 2015). These factors can further impact their neurodevelopment and aging later in life and will need to be carefully examined when studying neurodevelopmental trajectories. Interestingly, two studies have independently shown that while typically developing children show relations between brain volume and socioeconomic status, this effect is not seen in children with PAE (McLachlan et al., 2020; Uban et al., 2020). However, while both studies statistically controlled for the age of the children, neither study evaluated age interactions. Given the literature suggesting atypical brain trajectories for youth with PAE, it will be important to determine if socioeconomic status or other environmental factors may influence the brain trajectories.

Additionally, the majority of studies have examined brain development during childhood and adolescence, with comparatively fewer studies that have examined brain development in infants and toddlers or adults. The few studies that have applied MRI to examine the brain in infants and toddlers have shown that structural changes similar to what has been observed in children are present during this earlier developmental period, such as smaller corpus callosum (Jacobson et al., 2017), less gray matter (Donald et al., 2016), and changes in white matter microstructure (Donald et al., 2015). However, it is not known how brain development may change as a result of alcohol exposure during the infant and toddler period. To our knowledge, only one study has examined age–related changes in brain structure in adolescents and young adults. Inkelis et al. found that the relation between age and volumes of the corpus callosum, caudate, and cerebellum suggested that individuals with FASD may undergo an earlier brain degeneration than controls (Inkelis et al., 2020). Although this data analysis was cross-sectional and limited by its archival nature, it points towards a concerning possibility. Many studies have found atypical aging and early degeneration in other neurodevelopmental disorders characterized by both cognitive deficits and facial dysmorphology (e.g., Down, Prader-Willi, and Williams syndromes; Teipel and Hampel, 2006; Dykens, 2013; Zigman, 2013). To elucidate this question of atypical aging, longitudinal studies that follow individuals with PAE into later stages of adulthood are essential. To date, only a handful of studies have used MRI to examine the brain in young adults with PAE (reviewed in Moore and Riley, 2015), and even fewer have evaluated the interaction between age and exposure history.

Lastly, it will be important to study how the atypical neurodevelopmental trajectories relate to cognitive and behavioral deficits in FASD. Few studies have linked neurodevelopmental trajectories to cognitive and behavioral deficits in individuals with PAE. Further, it is unclear if cognitive or behavioral deficits may change as individuals with PAE mature.

## Conclusion

Individuals with FASD appear to have atypical cortical trajectories, at least in terms of cortical volume, thickness, and gyrification. For the majority of studies that noted differences in trajectories between individuals with PAE and controls, the general pattern was for those with PAE to have either blunted developmental change as compared to controls, which may indicate reduced neuroplasticity as compared to their typically developing peers, or accelerated declines with age that exacerbated group differences. These atypical brain trajectories suggest that behavioral and cognitive deficits may differ across age as well. Indeed, a recent cross-sectional analysis of the relation between cognition and age in children with PAE indicated that children with PAE may have slower gains in spatial working memory over time as compared to controls, resulting in a bigger gap in abilities by adolescence (Moore et al., 2021). However, other cross-sectional studies have failed to find differences in cognitive deficits between young and older children with PAE (Panczakiewicz et al., 2016). More research on the relationsip between cognition/behavior and age in those with PAE is needed, using longitudinal methodology ideally.

Cross-sectional studies indicate potential atypical subcortical development, specifically within the caudate, putamen, and amygdala in children, and within the caudate, cerebellum, and corpus callosum in adults. However, a large longitudinal study did not see differential age-trajectories in children and adolescents with PAE. More research is needed to determine if subcortical trajectories may differ for those with PAE. In particular, longitudinal studies across a wider age range would be informative. The cross-sectional studies examining subcortical trajectories utilized scans from children between ages 6 and 17, individuals 5–32, or adolescents and adults between ages 13 and 30, while the longitudinal study examined children aged 7–17. The 10-year age-range for the longitudinal study was relatively more restricted as compared to two of the three cross-sectional studies, which observed subcortical structure across 17–27 years. Thus, it is possible that a wider age range is necessary to be able to observe the developmental change in subcortical structures.

In terms of white matter trajectories, there is more convincing evidence of atypical trajectories at the microstructural level as compared to the gross measure of volume. Generally speaking, developmental change that was present for typically developing children was either absent or blunted for those with PAE. Although white matter change was attenuated for those with PAE, when white matter changes did occur, increases with age-predicted cognitive function in those with PAE; a relation that was not seen in typically developing individuals. Perhaps this finding may indicate that interventions aimed at improving white matter development could potentially be beneficial for those with FASD.

Only one longitudinal functional MRI study to date has been completed, and the results indicate that children with PAE may have atypical trajectory of brain processes underlying visuospatial attention. Again, this study suggested that individuals with PAE may have attenuated developmental change over time. However, more research is needed to understand the trajectory of functional brain processes underlying other aspects of cognition.

Few researchers have examined additional factors that may relate to these brain developmental trajectories in FASD. Thus far there is some evidence that there may be different developmental trajectories for males and females with PAE, and for individuals with such exposure who also exhibit facial dysmorphism. However, there are numerous other factors that may impact brain trajectories that have not yet been examined in those with PAE. Continued evaluation of neurodevelopmental trajectories in this population will be important, as such studies can assist in understanding pathology and windows for targeted interventions.

## Author Contributions

EM conceptualized the review. EM and YX both contributed to writing and editing the manuscript. YX produced the tables. All authors contributed to the article and approved the submitted version.

## Conflict of Interest

The authors declare that the research was conducted in the absence of any commercial or financial relationships that could be construed as a potential conflict of interest.

## Publisher’s Note

All claims expressed in this article are solely those of the authors and do not necessarily represent those of their affiliated organizations, or those of the publisher, the editors and the reviewers. Any product that may be evaluated in this article, or claim that may be made by its manufacturer, is not guaranteed or endorsed by the publisher.
